# Midkine and pleiotrophin in glioma: From mechanistic insights to therapeutic potential

**DOI:** 10.1016/j.neo.2026.101304

**Published:** 2026-04-08

**Authors:** Mieszko Lachota, Katarzyna Zielniok, Radosław Zagożdżon

**Affiliations:** Laboratory of Cellular and Genetic Therapies, Medical University of Warsaw, Warsaw, Poland

**Keywords:** Glioma, Glioblastoma, Midkine, Pleiotrophin, Tumor microenvironment, Cytokines

## Abstract

Midkine (MK) and pleiotrophin (PTN) are heparin-binding cytokines with growth factor properties that play essential roles in central nervous system development and tissue repair. Through pleiotropic, receptor-mediated signaling, they regulate fundamental cellular processes including survival, proliferation, migration, and stress adaptation. In cancer, these developmental programs are frequently co-opted to support tumor growth, angiogenesis, immune evasion, and microenvironmental remodeling via pathways such as PI3K/AKT, MAPK, and ALK.

In gliomas, both MK and PTN are consistently overexpressed, with expression increasing alongside tumor grade in IDH1 wild-type tumors, correlating with poor patient survival. Beyond direct tumor-cell effects, accumulating evidence indicates that MK and PTN shape the glioma microenvironment by promoting macrophage recruitment and polarization, modulating immune signaling, and influencing vascular remodeling.

This review synthesizes current knowledge on the molecular and cellular functions of MK and PTN in glioma biology, with particular emphasis on their partially overlapping yet distinct receptor and signaling networks that govern tumor cell survival, metabolic adaptation, and invasion. We outline their potential as therapeutic targets, discuss emerging ligand- and receptor-directed strategies, and identify key gaps that must be addressed to enable effective therapeutic translation, especially in light of the complementary and compensatory functions of these two cytokines.

## Introduction

Midkine (MK) and pleiotrophin (PTN) are soluble, secreted heparin-binding cytokines with growth factor properties that promote cell proliferation and migration [[1], [2], [3], [4], [5]]. They share approximately 50% structural homology, with similar terminal domains and comparable binding affinity to heparin and glycosaminoglycans [1,2,6,7]. Both MK and PTN play diverse roles in cell proliferation, inflammation, angiogenesis, oncogenesis, and stem cell self-renewal [[5], [6], [7], [8], [9], [10]].

In the healthy human central nervous system (CNS), MK and PTN are primarily produced by fetal astrocytes during neurogenesis [11,12]. Then, their expression progressively decreases to low levels in adults [13,14]. However, there is convincing evidence that both MK and PTN exert neuroprotective effects against drug-induced neurotoxicity and modulate the development of drug-induced neurodegenerative disorders [15]. They play a role in wound healing and are conditionally expressed following tissue injury, inducing cell proliferation, angiogenesis, monocyte chemotaxis, and chemokine expression [16,17]. However, these same growth- and proliferation-promoting properties can be hijacked in cancer [16]. Both MK and PTN are broadly expressed across many malignancies and have been functionally linked to poor prognosis due to their mitogenic, migratory, proinflammatory, and angiogenic effects [7,18]. Among malignancies in which MK and PTN have been implicated, gliomas are of particular interest [7].

Gliomas are the most common malignant primary CNS tumors in adults, with high-grade gliomas representing the leading cause of cancer-related death among patients aged 15–34 [19,20]. In WHO grade IV IDH^wild-type^ gliomas (formerly termed glioblastoma multiforme), standard-of-care therapy—consisting of maximal safe resection followed by temozolomide-based chemoradiotherapy—yields a median overall survival (OS) of approximately 16 months, a figure that has not improved in over 20 years [[19], [20], [21], [22]]. Although targeted therapies have transformed treatment in many cancers, only a small subset (1–5%) of gliomas can benefit from targeted therapies, e.g. tumors harboring BRAF p.V600E mutations may respond to BRAF inhibition. Overall, there is an urgent need for more effective treatment [23,24].

Both MDK and PTN are overexpressed in gliomas compared to healthy brain tissues [18,[25], [26], [27], [28]]. High expression of either cytokine correlates with poor prognosis, while combined high expression of both is associated with even worse outcomes [25]. Accumulating evidence indicates that MK and PTN promote glioma pathogenesis both through direct effects on tumor cells and through reprogramming of the tumor microenvironment [18,29,30]. Consequently, the MK-PTN axis represents a promising therapeutic target for standalone as well as combination therapy with cell-based approaches, due to their dual potential to both exert antitumor effects and modulate the microenvironment. This review summarizes current knowledge on the biological roles of MK and PTN in gliomas and outlines future directions for their development as biomarkers and therapeutic targets.

## Receptors and physiological functions

**Fig. 1:**
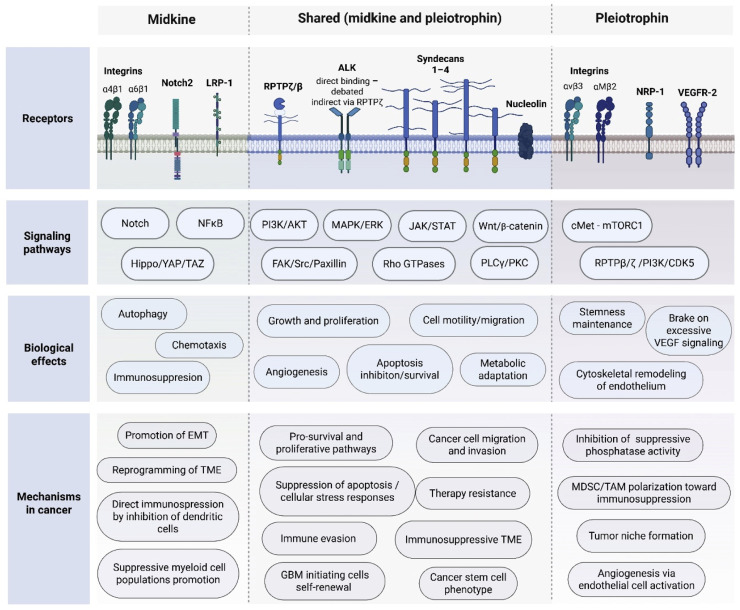
An overview of shared and distinct receptors, signaling pathways, and functional effects of midkine (MK) and pleiotrophin (PTN) in glioma.

**Fig. 2:**
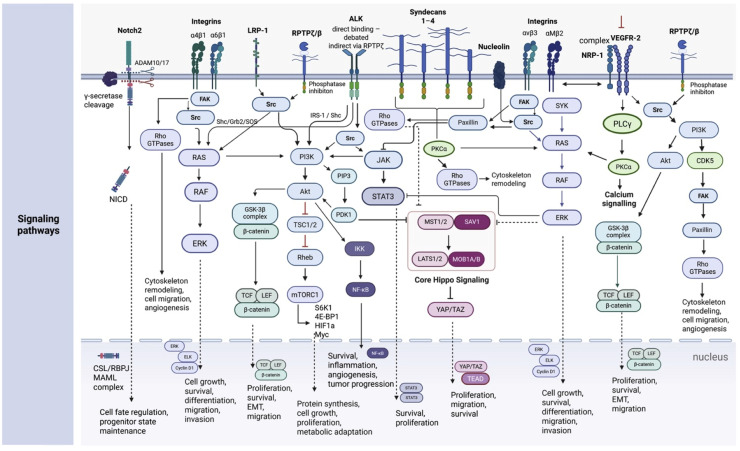
Receptor interactions and downstream signaling networks of midkine (MDK) and pleiotrophin (PTN). Midkine (MDK) and pleiotrophin (PTN) signal through a complex network of membrane receptors and co-receptors, leading to activation of multiple intracellular pathways that regulate cell fate, proliferation, survival, migration, and tumor progression. Both ligands interact with several cell-surface receptors, including receptor protein tyrosine phosphatase ζ/β (RPTPζ/PTPRZ1), anaplastic lymphoma kinase (ALK), syndecans, nucleolin, and integrins, while additional receptor interactions show ligand preference. MDK has been reported to signal through integrins α4β1 and α6β1, LRP1, and Notch2, whereas PTN preferentially engages integrins αvβ3 and αMβ2 and can signal in association with the neuropilin-1 (NRP1)–VEGFR2 receptor complex. Ligand binding induces receptor clustering and activation of multiple proximal signaling mediators, including Src family kinases, focal adhesion kinase (FAK), spleen tyrosine kinase (SYK), and adaptor proteins such as IRS-1 or Shc. In the case of PTN and MDK signaling via RPTPζ, ligand binding inhibits the phosphatase activity of the receptor, resulting in increased tyrosine phosphorylation of downstream signaling proteins, particularly Src family kinases. These proximal events converge on several central signaling hubs, including the RAS–MAPK pathway, PI3K–AKT signaling, and JAK–STAT3 activation. Downstream of PI3K–AKT, multiple pathways contribute to cellular growth and metabolic adaptation, including inhibition of the GSK3β destruction complex and stabilization of β-catenin, activation of mTORC1 signaling, and induction of NF-κB–dependent transcription. Concurrently, integrin- and Src-dependent focal adhesion signaling involving FAK and paxillin regulates cytoskeletal dynamics, cell migration, and invasion through activation of Rho family GTPases. PTN signaling through integrin αMβ2 can additionally activate SYK-dependent signaling cascades. Several transcriptional programs are activated downstream of these pathways. MAPK signaling induces ERK-dependent transcription through ELK family factors, while β-catenin accumulation promotes TCF/LEF-mediated gene expression associated with proliferation and epithelial–mesenchymal transition (EMT). Activation of STAT3 contributes to pro-survival and proliferative transcriptional programs, and the Hippo pathway modulates YAP/TAZ-dependent transcription controlling cell growth and migration. In addition, MDK-mediated activation of Notch2 results in nuclear translocation of the Notch intracellular domain (NICD) and formation of the CSL/RBPJ–MAML transcriptional complex, regulating cell fate determination and maintenance of progenitor states. Collectively, these signaling pathways illustrate substantial crosstalk and convergence downstream of MDK and PTN receptors.

The expression of midkine (MK) and pleiotrophin (PTN) is increased during fetal development, tissue injury, and in many human malignancies [6]. Both cytokines are potent heparin-binding growth factors involved in neuron development, tissue repair, inflammatory responses, and cancer [7,[31], [32], [33], [34], [35]]. Their pleiotropic roles include promotion of inflammatory cell recruitment, induction of chemokine expression, regulation of cell differentiation and growth, and mediation of anti-apoptotic effects [7,[31], [32], [33], [34], [35], [36], [37]]. Understanding the diversity of MK and PTN effects requires knowledge of their receptor repertoire, which can be broadly divided into proteoglycan and non-proteoglycan receptors. While beyond the scope of this glioma-focused review, we invite the readers to study multiple excellent reviews on the role of MK and PTN in human physiology and disease [7,31,34,35]. A schematic overview of MK and PTN receptors and their functions is presented in Fig. 1, while a detailed overview of the downstream signalling networks of MK and PTN is illustrated in Fig. 2.

### Proteoglycan receptors

The best-characterized receptors for midkine (MK) and pleiotrophin (PTN) are cell-surface proteoglycans, particularly receptor-type protein tyrosine phosphatase ζ/β (RPTPζ/β; *PTPRZ1*) and the syndecan family (Sdc1–4) [[38], [39], [40]]. Both MK and PTN bind these heparin- and chondroitin-sulfate–containing molecules with high affinity.

PTPRZ1 (RPTPζ/β) is physiologically expressed in the central nervous system, predominantly by glial cells and neural progenitors, and contributes to neuronal development, migration, and axon guidance [41,42]. During brain development, PTPRZ1 participates in neurite outgrowth through interactions with extracellular ligands including pleiotrophin and midkine [2,3]. In gliomas, however, PTPRZ1 expression is frequently elevated and enriched in glioma stem-like cells, where it promotes tumor cell proliferation, migration, and maintenance of stemness programs [4,5].

PTN binding to RPTPζ/β suppresses its phosphatase activity, leading to sustained activation of downstream tyrosine kinases such as anaplastic lymphoma kinase (ALK) [43]. Although direct MK/PTN–ALK binding remains debated, the MK/PTN–RPTPζ–ALK axis represents a central signaling module across neuronal and tumor models [[43], [44], [45]].

Syndecans, which display tissue-specific expression patterns, likely serve as co-receptors modulating MK/PTN availability and signaling [40,46]. In the healthy brain, syndecan family members show distinct spatial and developmental expression patterns. Syndecan-2 and syndecan-3 are the predominant neuronal isoforms and play key roles in neuronal maturation, dendritic spine formation, synaptic plasticity, and axon guidance [[47], [48], [49], [50], [51]]. Syndecan-3 is primarily localized to axons, whereas syndecan-2 is enriched at synapses [52]. During early brain development, syndecan-1 is expressed at high levels, particularly in ventricular regions containing proliferating neural precursor cells [53]. In gliomas, high expression of syndecan-1, syndecan-2, and syndecan-4 has been associated with tumor-promoting effects [[54], [55], [56]].

### Non-proteoglycan receptors

Beyond proteoglycans, MK and PTN also engage integrins and other noncanonical receptors. MK binds α4β1 and α6β1 integrins, while PTN interacts with αvβ3 and αMβ2 integrins to promote endothelial cell migration and angiogenesis [[57], [58], [59]]. PTN also binds with vascular endothelial growth factor receptor 2 (VEGFR2) and neuropilin-1 (NRP-1) mediating cell motility and regulating angiogenesis [58,60]. MK additionally binds to low-density lipoprotein receptor–related protein 1 (LRP1) and Notch2, with LRP1 signalling promoting macrophage survival and inflammatory signaling [61,62]. Both MK and PTN can also interact with nucleolin (NCL) on the cell surface, a lower-affinity binding that contributes to endothelial migration and survival [58,[63], [64], [65]].

## Expression, regulation, and clinical significance midkine

The role of midkine (MK) in gliomas gained attention after Mishima et al. first demonstrated in 1997 that *MDK* mRNA and protein levels are markedly elevated in high-grade astrocytomas, such as anaplastic astrocytoma and glioblastoma, compared with low-grade astrocytomas and normal brain tissue [28]. Subsequent in situ hybridization confirmed strong MK expression in most glioblastoma and low in a subset of low-grade astrocytomas [28]. Subsequent studies corroborated that MK expression correlates with tumor grade and exceeds that of healthy adult CNS tissue [18,[25], [26], [27]]. A recent analysis of a combined bulk RNA-seq dataset of 1017 gliomas, stratified by IDH status, further refined this relationship: *MDK* expression correlated with grade only in IDH-wild-type tumors, while IDH-mutant gliomas showed no consistent pattern [18]. The vast majority of MK is produced by glioma cells [18]. Moreover, several reports indicate that MK levels in gliomas are sufficiently high to elevate serum MK, suggesting its potential as a non-invasive biomarker [66,67]. Our ongoing study confirms this observation, showing significantly increased MK serum concentrations in glioblastoma patients at diagnosis compared with healthy donors (1082pg/ml vs 463pg/ml, *p* < 0.0001; Lachota et al., unpublished).

There are multiple mechanisms driving MK overexpression in gliomas, however, their significance remains incompletely understood. Originally identified as a retinoic acid–inducible gene [1,12], MK may be upregulated in gliomas through aberrant retinoic acid signaling, which is commonly overactive in high-grade tumors and linked to proliferation and poor prognosis [68]. Additional regulatory inputs include Wnt/β-catenin signaling, as Wnt3a administration or β-catenin activation increases MK expression via a TCF/LEF site in the *MDK* promoter [69], and hypoxia, which induces MK through hypoxia-responsive elements [70].

The *MDK* promoter also contains an SP1-binding site that positively regulates MK transcription: silencing either *MDK* or SP1 suppresses glioma proliferation and tumor growth, whereas MK overexpression rescues the SP1-silenced phenotype [71]. DNA-damage-induced p53 [72] and copy-number gains at 11p11.2 (the *MDK* locus) [73] further contribute to overexpression. In non-glioma tissues, estrogen signaling was shown to modulate MK through ER-β–PKCδ activation [74,75] and ER-α repression [76], while glucocorticoid receptor activity suppresses MK [77]. Additionally, in murine NF1-associated optic pathway gliomas, neuronal hyperexcitability induces MK, and suppression with lamotrigine lowers its expression [78]. Interestingly, lamotrigine treatment has been associated with longer progression-free survival in high-grade glioma, though MK involvement was not directly examined [79]. At the post-transcriptional level, MK is regulated by N^6^-methyladenosine (m^6^A) modification. The m⁶A regulators *HNRNPA2B1, HNRNPC*, and *WTAP* are upregulated in glioblastoma, potentially driving protumorigenic signaling via the MK/PTN and Galectin signalling [80]. Finally, cytokines regulate MK expression: TNF-α, EGF, and FGF-10 all enhance MK production in monocytes and tumor cells [[81], [82], [83], [84]].

High MK expression correlates with poor overall survival [18,25,27,85]. Moreover, MK is linked to high WHO grade, IDH^wild-type^ status, low KPS score, and shorter recurrence interval, as well as adverse gene expression signatures, 1p/19q non-codeletion, and unfavorable methylation profiles [18,25,70,[85], [86], [87], [88], [89], [90], [91], [92]]. While MK expression was investigated as a predictive biomarker for anti-VEGF therapy (bevacizumab ± temozolomide) and for crizotinib + temozolomide combinations, no significant associations with treatment response were found [93].

### Pleiotrophin

Pleiotrophin (PTN) exists in two isoforms (18 kDa and 15 kDa), both found in gliomas [94,95]. PTN mRNA and protein are ubiquitously detected in grade III–IV gliomas, primary glioma cell cultures, and established cell lines, as shown by both *in situ* and *in vitro* studies [25,[96], [97], [98], [99], [100], [101]]. Immunohistochemistry on 78 primary CNS tumors revealed that PTN expression is significantly higher in glioblastoma and anaplastic astrocytoma than in pilocytic or diffuse astrocytoma, but shows no difference between oligodendroglioma grades. No association between *PTN* expression and Ki67, microvascular density and overall survival was found [102]. Recent work identified a circular RNA form of pleiotrophin (circ_PTN), a circular RNA derived from the PTN locus, which is likewise upregulated in glioma cells [103,104].

Like MK, PTN expression can elevate serum PTN levels, which are significantly higher in glioma and other cancers than in healthy controls [66]. Serum concentration of both MK and PTN also moderately correlate (R^2^ = 0.546) [66]. Their shared receptors, including RPTPζ/β and ALK, are also upregulated in glioma tissues and cell lines [98,99,101,105], with ALK particularly abundant in aberrant, poorly perfused tumor vasculature [101].

PTN is transcriptionally regulated by a combination of developmental and stress-responsive factors, including MYOD1, trihelix transcription factor GT-1, activator protein 1 (AP-1), serum homeobox A5 (HOXA5), Sox10, and PDGF signaling [100,106,107]. Additional control by NF-κB, CREB, and serum response factor reflects its sensitivity to inflammatory and mitogenic stimuli [35,108,109]. In contrast to MK, PTN expression is not linked to retinoic acid signalling [77,106,108].

Inflammatory cytokines—TNF-α, IFN-β, and IFN-γ—robustly stimulate PTN production in monocytes and tumor cells [81,83,84,110,111]. Growth factors such as EGF, FGF-10, FGF2, and PDGF enhance PTN expression, whereas VEGF-A suppresses it [81,82,106,[112], [113], [114]]. In contrast to MK, sex hormones (progesterone, testosterone) induce PTN [115], while glucocorticoid receptor signaling inhibits both [77,116].

PTN is also produced by tumor-associated macrophages (TAMs), stimulating glioma stem cells through PTPRZ1 [117,118]. Its expression in antigen-presenting cells depends on nucleic-acid–sensing TLRs (3, 7, 8, 9), and stimulation of these receptors consistently induces PTN—at higher levels than MK—across innate APC subsets [117,118].

Clinically, PTN expression correlates with higher WHO grade, low KPS, shorter time to recurrence, and poor overall survival [25,96,101]. Co-expression of MK and PTN confers an even poorer prognosis [25], and in recurrent glioblastoma, PTN overexpression serves as an independent prognostic factor [119].

Midkine and pleiotrophin both bind heparin and related glycosaminoglycans, which can modify their availability and activity. N-Acetylgalactosamine 4-sulfate 6-O-sulfotransferase (GalNAc4S-6ST) is an enzyme catalyzing the synthesis of the highly sulfated chondroitin sulfate motif CS-E that is ubiquitously expressed in gliomas. GalNAc4S-6ST mRNA expression has been reported as an independent risk factor associated with poor prognosis [120]. In Boyden-chamber assays, CS-E enhanced migration of U251-MG glioblastoma cells toward CS-E’s preferred ligands, pleiotrophin (PTN) and midkine (MK) [120]. Another regulator is apican, a chondroitin-sulfate proteoglycan secreted by C6 glioma cells that contains an E disaccharide (–GlcUA-GalNAc(4,6-O-disulfate)–) in its chondroitin sulfate (CS) chain. C6-derived apican was shown to bind both MK and PTN, whereas apican CS from SH-SY5Y neuroblastoma cells (which lacked the E disaccharide) did not, suggesting that apican CS may play a role fine-tuning MK- and PTN-mediated protumorigenic effects in glioma [121].

**Fig. 3:**
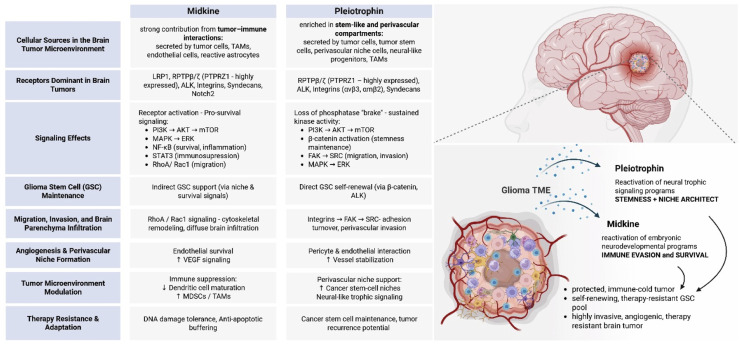
Distinct and complementary roles of midkine (MK) and pleiotrophin (PTN) in shaping glioma cell behavior and the tumor microenvironment. The schematic summarizes their major cellular sources in brain tumors, their receptors, downstream signaling effects, and functional consequences for glioma stem cell maintenance, invasion, angiogenesis, immune modulation, and therapy resistance, highlighting MK-driven immune evasion and survival buffering and PTN-driven stemness and perivascular niche support.

Collectively, the coordinated upregulation of MK, PTN and their modifing factors in gliomas reflects the convergence of developmental, metabolic, and inflammatory programs co-opted by tumor cells and their microenvironment. Acting as partially parallel mediators within a shared stress-response network, these ligands translate diverse oncogenic and environmental cues into pro-tumorigenic signals (Fig. 3).

### Proliferation, survival, and migration

#### Midkine

MK promotes glioma cell proliferation and survival primarily through either direct or indirect activation of ALK and its downstream pathways. In U87MG cells, ALK reduction abolishes MK-induced Akt phosphorylation, highlighting the rate-limiting role of ALK in MK signaling [44]. Recent bioinformatic analyses combined with partial experimental validation have pointed towards MK involvement in JAK–STAT, cell cycle progression, and VEGF signaling [70,85].

MK signalling has been implicated in glioma cell migration, although available findings are not fully concordant. Early Boyden chamber assays detected no chemotactic response to MK [122], whereas subsequent studies reported elevated MK expression in highly migratory U373MG clones and primary GBM cells [123], and demonstrated that MK overexpression enhances basal migration and invasiveness while its knockdown suppresses these properties [85]. These discrepancies arise from differences in experimental design—specifically, assays measuring directional chemotaxis versus those assessing baseline motility or invasiveness. Taken together, the available evidence suggests that MK primarily enhances intrinsic migratory and invasive capacity of GBM cells rather than acting as a direct chemotactic cue.

Beyond proliferation and migration, MK contributes to metabolic adaptation. Extracellular acidosis is a hallmark of gliomas, and MK supports survival under low pH via acyl-CoA synthetase 5 (ACSL5) [124]. Hypoxia further upregulates MK through hypoxia-responsive elements in its promoter, promoting proliferation, migration, and EMT under hypoxic conditions [70]. This potentially impacts angiogenesis and vessel integrity, increasing the risk of intracranial hemorrhage [125].

Resistance to autophagic cell death is also affected by MK. Combined treatment of glioma cells with imatinib and noscapine or lithium chloride paradoxically induced proliferation, protecting cells from autophagy-mediated death due to increased MK secretion [126,127]. MK has been linked to resistance to Δ9-tetrahydrocannabinol (THC)-induced autophagy, with its silencing or ALK inhibition restoring sensitivity [128].

Overall, in glioma, MK overexpression enhances cell proliferation, survival, and invasion whereas its suppression produces the opposite effects [26,70,85].

#### Pleiotrophin

PTN signaling parallels that of MK, primarily through ALK-mediated Akt activation [44]. In xenograft models, PTN knockdown modestly decreased U87 proliferation, whereas combined PTN/ALK knockdown abolished tumor growth [129], suggesting compensatory MK activity. PTN effects appear to be isoform-dependent [95,130].

PTN has been implicated in glioma cell migration, yet the literature is characterized by apparently conflicting findings. Immobilized 18 kDa PTN induces RPTPβ/ζ-dependent haptotactic migration [95], and subsequent studies confirmed weak chemotactic but strong haptotactic responses through RPTPβ/ζ [98,99]. In contrast, other reports detected no chemotactic effect of either PTN or MK across multiple glioblastoma lines [122]. Moreover, PTN has been shown to inhibit migration in cells expressing RPTPβ/ζ alone, while stimulating migration in U87MG cells co-expressing RPTPβ/ζ and αvβ3 integrin [131]. Mechanistic work by Koutsioumpa et al. reconciled these discrepancies by demonstrating that PTN-induced migration requires coordinated surface expression of RPTPβ/ζ, αvβ3, and nucleolin. In this model, PTN signaling through RPTPβ/ζ and c-Src promotes αvβ3-dependent β3 phosphorylation, enabling nucleolin translocation to the cell surface and formation of a functional promigratory receptor complex [132]. Together, these data indicate that PTN’s migratory effects are highly receptor-context dependent, explaining why earlier studies reported divergent outcomes. Additionally, the authors have also shown that cell surface expression of NCL highly correlates with αvβ3 expression in primary human glioblastoma, with the correlation coefficient rising proportionally to the tumor grade, likely increasing the susceptibility of the high grade tumors to proinvasive effects of PTN [132].

PTN may also influence chromosomal stability and proliferation, as dominant-negative PTN induces tetraploidy and G1 arrest [133]. PTN overexpression is associated with chromosome 7 amplification in the early glioblastoma development [134]. Circ_PTN, a circular RNA derived from PTN, regulates glioma proliferation, cell cycle, apoptosis, and glycolysis via miR-122/SOX6 [103,104]. Additionally, PTN likely contributes to autophagy resistance through ALK activation, although direct mechanistic evidence is limited [128,135].

Collectively, available experimentally-validated evidence suggests that MK and PTN converge on ALK-dependent PI3K–Akt signaling to promote proliferation and survival, while context-specific engagement of other receptors determines e.g. their migratory effects. However, much is unknown about the role of other receptors that are involved in MK and PTNs complex signaling architecture. Future studies should determine which receptors are expressed and necessary for glioma-promoting effects, whether MK and PTN act redundantly or complementarily, and whether concurrent inhibition of both is necessary to disrupt this signaling network effectively.

### Glioma-initiating cells

Glioma-initiating cells (GICs) constitute a self-renewing subpopulation capable of producing and responding to their own growth factors, thereby sustaining tumor propagation and treatment resistance in glioblastoma.

Early studies established the PTN–RPTPβ/ζ–ALK axis as essential for glioblastoma stem cell self-renewal and tumorigenicity [136,137]. Further, MK was identified as a key factor secreted by sphere-forming GICs, promoting proliferation, survival, and protection from reactive oxygen species (ROS) in both autocrine and paracrine fashions. Neutralizing MK with specific antibodies reduced GIC sphere survival by inducing cell-cycle arrest and apoptosis secondary to oxidative stress–induced DNA damage [138]. Sensitivity to MK inhibition varied among GICs and was determined by PCBP4, a p53-inducible protein that predicts resistance to anti-MK therapy. Because p53 activation also upregulates MK in response to DNA damage, it is plausible that a subset of p53-driven, MK^high^ GICs exhibits intrinsic resistance to MK blockade. Interestingly, despite shared receptors and downstream pathways, PTN was not implicated as a resistance determinant.

Targeting MK or its effector ALK with neutralizing antibodies or small-molecule inhibitors (crizotinib, lorlatinib) suppresses GIC self-renewal and tumorigenicity by inducing autophagic degradation of the transcription factor SOX9, additionally sensitizing GICs to temozolomide [139]. Consistent findings demonstrated that MK enhances temozolomide resistance and stem-like properties in glioma cells via Notch1/p-JNK signaling, correlating with elevated Notch1, p-JNK, and CD133 expression in tumor specimens. *MDK* knockdown markedly inhibited tumor growth in xenograft models [140].

Phenotypic shifts toward stem cell–like states after therapy are recognized drivers of glioblastoma recurrence. It is possible that the reason may be the development of a tumorigenic milieu in the resection margin [119,141]. In an orthotopic xenograft resection model with integrated 18F-FET PET/CT and transcriptomic profiling, recurrent tumors were enriched in signatures of microglia/macrophage infiltration, angiogenesis, and stem-cell activation. PTN, normally involved in tissue regeneration, was markedly elevated in recurrent lesions and proposed as the key mediator of this iatrogenic stemness induction [119].

PTN is also enriched in specific neurogenic regional niches. In the healthy adult brain, PTN expression is low overall but maintained in the subventricular zone (SVZ), where neural precursor cells (NPCs) physiologically support tissue regeneration. The SVZ is a frequent site of glioma infiltration, and PTN secreted by resident NPCs has been shown to attract glioma cells and provide a permissive niche for their expansion, as demonstrated by in vivo PTN knockdown and spatial expression analyses [142]. In apparent contrast, an ACNU-resistant glioma subline exhibiting stem cell–like features showed reduced PTN expression [143], while MK levels were not assessed. This finding suggests that PTN may not be uniformly required for maintenance of all therapy-resistant stem-like states, and that its role in glioma stemness may depend on treatment context, compensatory MK signaling, or microenvironmental support.

Tumor-associated CD11b⁺/CD163 macrophages (TAMs) also secrete PTN, stimulating GICs via RPTPβ/ζ. Co-implantation of M2-like macrophages augmented GIC-driven tumor growth, whereas silencing PTN in these macrophages abrogated their protumorigenic activity. The receptor RPTPβ/ζ is preferentially expressed in GICs and associates with adverse outcome, while its inhibition by shRNA or blocking antibodies suppresses glioma growth [137].

Together, these findings support a model in which MK and PTN help establish a stemness-supportive niche spanning multiple cellular compartments—GICs, NPC-rich neurogenic regions, and infiltrating macrophages—that reinforces self-renewal and therapeutic resistance. A key outstanding challenge is to define which ligand–receptor interactions are truly required to maintain the GIC state in vivo and how these dependencies shift following radiotherapy or surgical injury. Clarifying these dynamics will be essential for both designing and proper timing of the MK- and PTN-targeting interventions.

## Tumor microenvironment interactions

The tumor-promoting activity of MK extends beyond its direct effects on glioma cells. Early studies suggested that MK interacts with RPTPβ/ζ on endothelial cells, modestly reducing migration and thus potentially modulating angiogenic balance [131].

Subsequent research has revealed that MK promotes macrophage recruitment and drives them towards a immunosuppressive, pro-tumorigenic phenotype [80]. These findings align with results from Zhang et al.[30], who demonstrated that in gallbladder cancer MK binds to low-density lipoprotein receptor-related protein 1 (LRP1) on infiltrating macrophages, triggering an immunoregulatory transcriptional program that reinforces the suppressive TME. Consistent with these data, a recent study supports these findings showing that *MDK*^high^ tumors exhibited upregulation of more than 20 chemokines**,** including MIP-1a (CCL3), CCL13, CCL14, CCL17, CCL18, CXCL5, CXCL6, CXCL10, CXCL11, cytokines interleukin 1 alpha (IL-1α), interleukin 10 (IL-10), interleukin 15 (IL-15), interleukin 33 (IL-33), interferon γ (IFN-γ), granulocyte-macrophage colony-stimulating factor (GM-CSF), platelet-derived growth factor AA/BB (PDGF-AA/BB), and other soluble factors such as programmed death-ligand 1 (PD-L1), granzyme B [18]. Functional experiments confirmed the dominant role of MK in inducing expression of these soluble factors in macrophages, supporting a broader role for MK in sustaining an inflammatory yet immunosuppressive TME [18].

Evidence for direct effects of midkine (MDK) on T cells remains limited. Available studies suggest that MDK can influence T-cell responses by restricting regulatory T-cell differentiation in autoimmune models [144,145] and by enhancing T-cell activation and Th1 polarization through calcineurin–NFAT and IL-12/STAT4 signaling pathways [146]. In addition, neuron-derived MDK has been reported to stimulate naïve CD8⁺ T cells to produce CCL4 via LRP1-dependent signaling, establishing a neuron–T cell communication axis in glioma models [147]. To best of our knowledge, the role of PTN on T cell biology was not assessed.

The role of pleiotrophin (PTN) in the glioma microenvironment has been investigated primarily in the context of angiogenesis. PTN displays context-dependent, biphasic activity, capable of both promoting and restraining vascular growth depending on receptor availability and microenvironmental cues. In murine GL261 gliomas, PTN overexpression increased microvessel density and accelerated tumor growth; both effects were abrogated by inhibitors of ALK or VEGF signaling (crizotinib, ceritinib, cediranib), indicating a pro-angiogenic, pro-tumorigenic role [101]. Conversely, conditioned medium from PTN-silenced glioma cells enhanced endothelial proliferation, migration, and tube formation in a VEGF-dependent manner [148], suggesting that endogenous PTN may restrain certain VEGF-driven responses. Mechanistically, PTN competes with VEGF165 for binding to RPTPβ/ζ, attenuating VEGF-induced endothelial migration to levels comparable to its own weaker stimulus [149]. Thus, PTN displays biphasic, context-dependent activity: while overexpression can promote angiogenesis through ALK and VEGF pathway cooperation [101], endogenous PTN may simultaneously limit excessive VEGF signaling depending on ligand concentration, receptor availability, and microenvironmental stoichiometry.

Receptor context further modulates these effects. PTN mediates its pro-migratory activity through RPTPβ/ζ and αvβ3 integrin—but not α5β1—whereas midkine, although binding the same RPTPβ/ζ, fails to engage αvβ3, explaining its weaker effects. Accordingly, PTN inhibits migration in glioma lines expressing only RPTPβ/ζ but stimulates migration in U87MG cells co-expressing RPTPβ/ζ and αvβ3 [131]. Supporting a direct pro-angiogenic contribution, PTN also induces dose-dependent chemotaxis of endothelial progenitor and umbilical vein endothelial cells via nitric oxide– and PI3K-dependent mechanisms, with potency comparable to VEGF [150].

Overall, the data indicate that MK and PTN shape the glioma microenvironment through clearly defined, targetable mechanisms: MK drives macrophage recruitment and immunosuppressive reprogramming, while PTN modulates angiogenic output through receptor-dependent crosstalk with VEGF pathways. These signals converge to reinforce a microenvironment that supports invasion, stemness, and therapy resistance.

## Therapeutic targeting strategies

Because of their pleiotropic pro-tumorigenic effects, both MK and PTN have emerged as attractive yet challenging therapeutic targets (Table 1). Current strategies to counteract their activity include direct ligand inhibition or interference with receptor signaling.

**Table 1:** Therapeutic strategies targeting Midkine (MDK) and Pleiotrophin (PTN) signaling across intervention levels.

Ligand axis	*Target level*	*Strategy / agent*	*Molecular target*	*Mechanism*	*Evidence / disease context*	*Key outcomes*	*Main limitations*
MDK	Ligand targeting	Neutralizing anti-MK antibodies (incl. CAB-101/102)	MK	Ligand neutralization	Osteosarcoma and breast cancer murine models	Tumor suppression	No peer-reviewed data
MDK	Ligand targeting	HBS-101	MK	Ligand neutralization	Triple negative breast cancer *in vivo* and murine models	Tumor suppression	No clinical validation
MDK	Expression modulation	iMDK	MK	Small-molecule inhibition of MDK transcription and PI3K signalling	Orthotopic GL261 glioma; lung and oral SCC models	Reduced tumor growth, prolonged survival, decreased M2 polarization, anti-angiogenic effects	No clinical validation; BBB penetration uncertain
MDK	Expression modulation	Lamotrigine	Neuronal activity → MK expression	Suppression of activity-dependent MK upregulation	NF1-associated glioma models; retrospective high-grade glioma cohort	Reduced tumor growth in models; association with longer PFS	MK dependency not confirmed clinically
MDK	Expression modulation	Imatinib, roscovitine	MK production pathways	Reduced MK expression by GBM cells	In vitro GBM	Decreased MK levels	Indirect effect; BBB delivery concerns
MDK	Receptor / downstream	Crizotinib	ALK (MK/PTN-activated tumors)	Inhibition of MK/PTN downstream signaling	Phase I glioblastoma (with TMZ + RT)	PFS 10.7 mo; OS 22.6 mo; tolerable	No randomized control; target heterogeneity
MDK	Receptor / downstream	Ceritinib	ALK/IGF1R/FAK axis	Kinase inhibition	Phase 0 glioblastoma	No pharmacodynamic inhibition	Subtherapeutic intratumoral levels; BBB limitation
MDK	Gene targeting	Ad-MK (MK promoter-driven oncolytic adenovirus)	MK-high cells	Tumor-selective viral replication	Glioma xenografts	Eradication of MK-positive tumors	Delivery and safety considerations
MDK	Gene targeting	ROS-responsive liposomes (siRNA / CRISPR vs MK)	MK gene	Targeted gene silencing	Orthotopic GBM	Tumor growth inhibition	Stability and delivery efficiency
PTN	Ligand targeting	Anti-PTN–saporin conjugate; antibody 7E4B11	PTN	Ligand neutralization / toxin delivery	U87 glioma xenografts	Delayed tumor growth	Early preclinical stage
PTN	Receptor / downstream	Anti-ALK antibodies	ALK	Blockade of PTN/MK signaling	In vitro and U87MG xenografts	Reduced invasion and tumor growth	ALK not universally expressed
PTN	Gene targeting	Ribozymes vs PTN or ALK	PTN / ALK	Expression knockdown	GBM cell lines and xenografts	Reduced proliferation, migration, angiogenesis	Delivery barriers
PTN	Receptor targeting	PTPRZ1 knockdown (siRNA; CRM197-PEI)	PTPRZ1 (RPTPβ)	Silencing receptor enriched in GSCs	U251-MG xenografts	Near-complete tumor suppression	Nucleic acid delivery challenges
PTN	Engineered biologics	Dominant-negative PTNΔ111-136	PTN	Heterodimerization with WT PTN → functional neutralization	In vitro models	Reduced proliferation and angiogenesis	Experimental strategy
PTN	Engineered biologics	PTN97–110 peptide	PTN and VEGFA165 pathways	Anti-angiogenic signaling blockade	In vitro, CAM, zebrafish, glioma models	Reduced endothelial migration and vascularization	PK and delivery unknown
PTN	Expression modulation	Indatraline	Monoamine transporters → PTN/VEGF	Neurotransmitter-dependent suppression of PTN	In vitro GBM	Reduced migration, spreading, tube formation	Repurposing concept; no in vivo glioma data

Abbreviations: BBB, blood–brain barrier; GBM, glioblastoma; GSCs, glioma stem-like cells; MK, midkine; PTN, pleiotrophin; RT, radiotherapy; TMZ, temozolomide; VEGF, vascular endothelial growth factor.

The small-molecule *MDK* transcription inhibitor iMDK suppressed glioma progression in orthotopic GL261 models, reducing tumor growth, prolonging survival, and limiting M2 macrophage polarization through inhibition of MK/LRP1 signaling [151]. Similar anti-tumor and anti-angiogenic effects have been observed in additional solid tumor models [29,152,153], supporting broader applicability.

Recently, another MK-specific small-molecule inhibitor, HBS-101, has been described. HBS-101 binds midkine and disrupts its interaction with cognate receptors. In triple-negative breast cancer, it exhibited potent antitumor activity by suppressing the Akt/mTOR, STAT3, and NF-κB signaling pathways. Notably, HBS-101 demonstrated good oral bioavailability and the ability to penetrate the blood–brain barrier [154]. However, its effect on PTN was not reported.

Neutralizing anti-MK antibodies have demonstrated tumor-suppressive activity in breast cancer, osteosarcoma and hepatocellular carcinoma models [155,156]. Although no antibody therapies against MK or PTN have reached clinical testing, industry-developed candidates such as CAB-101 and CAB-102 have shown preclinical anti-cancer activity reported by the company (in a non-peer reviewed publication) [157].

In gliomas associated with NF1, neuronal hyperexcitability has been identified as a driver of MK overexpression. The anti-epileptic drug lamotrigine, which suppresses neuronal firing, reduced tumor growth by downregulating MK [78]. A subsequent large-scale analysis linked lamotrigine use to prolonged progression-free survival in high-grade glioma, though a role of MK remains unconfirmed [79].

Several kinase inhibitors—including imatinib and roscovitine (seliciclib)**—**reduce MK production by GBM cells [158]. However, clinical translation remains constrained by blood–brain barrier penetration, as illustrated by a phase 0 ceritinib trial that failed to achieve adequate intratumoral pharmacodynamic inhibition [159].

Targeting downstream effectors of MK signaling represents another promising approach. Although activating ALK mutations are uncommon in adult gliomas and ALK is not generally overexpressed**,** MK/PTN-high tumors are characterized by ALK activation [160]. The dual ALK/c-MET inhibitor crizotinib has shown activity when combined with standard therapy (temozolomide + radiotherapy), yielding median progression-free and overall survival times superior to typical outcomes [161,162]. In a phase I trial (NCT02270034), the regimen was well tolerated, though no direct control arm was included [67,139,[22], [163], [164], [165], [166], [167], [168], [169]].

Gene therapy approaches have exploited the potent activity of the *MDK* promoter in glioma. A midkine promoter–driven conditionally replicating adenovirus (Ad-MK) showed cytolytic activity specifically in MK-positive glioma cells, but not in normal brain cells. In vivo, E3-intact Ad-MK eradicated MK-positive xenografts, highlighting its promise for targeting chemoresistant, MK-high glioma stem cell populations [170]. Another recent work has utilized ROS-cleavable fusogenic liposomes carrying siRNA or CRISPR–Cas9 complexes against MK, achieving efficient gene silencing and tumor growth inhibition in orthotopic GBM models [171].

Although PTN has been less extensively explored, early results parallel those of MK. Anti-PTN antibodies conjugated to the ribosome-inactivating toxin saporin markedly delayed tumor growth in U87 xenografts, while the unconjugated antibody 7E4B11 alone provided modest, but significant benefit, confirming the intrinsic growth dependency of glioma cells on PTN [172]. Similarly, inhibition of MK/PTN signaling through anti-ALK antibodies blocked endothelial monolayer invasion and reduced growth of U87MG xenografts [87].

Molecular knockdown of PTN or ALK suppressed glioblastoma proliferation, migration, and angiogenesis *in vitro* and *in vivo* [105,173,174]. Small-molecule inhibitors such as crizotinib, ceritinib, and the VEGFR inhibitor cediranib also curtailed the growth of orthotopic gliomas derived from PTN-overexpressing, but not wild-type, GL261 cells [101]. As noted above, inadequate intratumoral drug exposure due to blood-brain barrier remains a key translational obstacle [159].

Direct targeting of MK and PTN shared receptor RPTPβ/ζ, overexpressed in glioma stem-like cells, produced potent anti-tumor effects in xenograft models, including near-complete tumor suppression following siRNA-mediated knockdown [175,176]. These findings support the feasibility of nucleic acid–based strategies, pending further optimization of delivery and stability.

Engineered PTN variants provide an additional strategy. A dominant-negative mutant can heterodimerize with wild-type PTN and inhibit glioma proliferation, tumor growth, and angiogenesis as shown in preclinical models [177]. Similarly, a PTN-derived peptide (PTN97–110) blocked PTN- and VEGFA165-mediated pro-angiogenic signaling and suppressed endothelial activation and tumor-associated angiogenesis *in vitro* and *in vivo* [149,178]. Such engineered ligands could be further adapted into modular immunotherapies—for example, CAR-T cells locally releasing them to remodel the tumor microenvironment.

Finally, pharmacological modulation of neurotransmitter signaling may indirectly downregulate PTN. The nonselective monoamine transporter inhibitor indatraline suppressed GBM cell migration, spreading, and tube formation while elevating Rho GTPase activity and reducing PTN and VEGF expression, pointing to a potential repurposing avenue for neurotransmitter-modulating agents in glioma [179].

Therapeutic targeting of MK and PTN in gliomas remains challenging due to their large receptor network, context-dependent and partially overlapping effects. Current strategies—ranging from ligand neutralization and receptor inhibition to promoter-driven gene therapies—show preclinical efficacy, yet it remains unclear whether optimal intervention requires targeting MK, PTN, their shared receptors, or selected combinations thereof. Blood–brain barrier penetration, tumor heterogeneity, and compensatory signaling further complicate translation. Overall, these approaches highlight promising avenues but underscore that fundamental questions as what to target, are still unresolved.

## Conclusions

Midkine (MK) and pleiotrophin (PTN) are emerging as central modulators of glioma biology, bridging tumor-intrinsic signaling and microenvironmental remodeling. Evidence indicates that MK/PTN sustain glioma proliferation, invasion, and stemness through ALK-dependent pathways while concurrently shaping a cytokine-rich niche that favors macrophage recruitment, T-cell dysfunction, and vascular remodeling. These dual roles—cell-autonomous support and non-cell-autonomous TME orchestration—underlie the association of MK/PTN expression with aggressive, therapy-resistant gliomas.

Despite its evident protumorigenic role, therapeutic targeting remains challenging. MK/PTN engage a redundant, context-dependent receptor network, making single-node inhibition prone to compensation. Dynamic regulation by hypoxia, neuronal activity, and post-surgical injury further complicates dosing and timing. Additionally, delivery constraints such as CNS penetration and intratumoral exposure pose another obstacle.

Recent advances in biologics offer potential solutions. Multi-epitope or dual-ligand nanobody constructs could neutralize both MK and PTN while achieving satisfactory penetration to CNS [180]. A perioperative window may be particularly amenable to intervention: surgical injury induces a transient niche that supports glioma stem-cell survival and early regrowth, which MK/PTN blockade could suppress. Optimizing timing, delivery route, and combination with standard therapies will be essential to leverage this window effectively. Safety considerations are encouraging, as adult baseline MK/PTN expression is minimal.

Beyond therapeutics, MK overexpression is strongly associated with glioblastoma grade and survival outcomes, and aligns with a mesenchymal–injury transcriptional program enriched in cytokines and chemokines. This reproducible signature may serve as both a prognostic marker and a predictive tool for patient selection for both MK/PTN specific and non-specific treatments.

In conclusion, MK and PTN define a promising actionable axis in glioblastoma. Utilizing their translational potential will require informed ligand/receptor blockade, CNS-optimized delivery, proper timing, and biomarker-driven patient stratification. If these constraints are addressed, MK/PTN axis could provide therapeutic opportunities for a large subset of high-grade glioma patients.

## Funding

Mieszko Lachota is supported by the Foundation for Polish Science under the START Programme.

## CRediT authorship contribution statement

**Mieszko Lachota:** Writing – review & editing, Writing – original draft, Conceptualization. **Katarzyna Zielniok:** Writing – review & editing, Writing – original draft. **Radosław Zagożdżon:** Writing – review & editing, Writing – original draft.

## Declaration of competing interest

R.Z. was employed by the company 4Cell Therapies S.A. (Gliwice, Poland) as an ad hoc Research Consultant. M.L., K.Z., and R.Z., have a patent pending “EP4538706 - NEW MARKERS FOR DIAGNOSING GLIOBLASTOMA MULTIFORME AND USES THEREOF”.
